# Priorities for improvement across cancer and non-cancer related preventive services among rural and non-rural clinicians

**DOI:** 10.1186/s12875-022-01845-1

**Published:** 2022-09-09

**Authors:** Michaela Brtnikova, Jamie L. Studts, Elise Robertson, L. Miriam Dickinson, Jennifer K. Carroll, Alex H. Krist, John T. Cronin, Russell E. Glasgow

**Affiliations:** 1 Adult and Child Consortium for Health Outcomes Research and Delivery Science (ACCORDS), University of Colorado School of Medicine and Children’s Hospital Colorado, 1890 N. Revere Court, Mailstop F443, Aurora, CO 80045 USA; 2 Department of Pediatrics, University of Colorado School of Medicine, Aurora, CO USA; 3 Division of Medical Oncology, Department of Medicine, University of Colorado School of Medicine, Aurora, CO USA; 4 University of Colorado Cancer Center, Aurora, CO USA; 5 American Academy of Family Physicians National Research Network, Leawood, KS USA; 6 Department of Family Medicine, University of Colorado School of Medicine, Aurora, CO USA; 7 Department of Family Medicine and Population Health, Virginia Commonwealth University, Richmond, VA USA

**Keywords:** Cancer prevention and control, Screening, Rural, Primary care, Need for improvement

## Abstract

**Introduction:**

It is not realistic for most clinicians to perform the multitude of recommended preventive primary care services. This is especially true in low resource and rural settings, creating challenges to delivering high-quality care. This study collected stakeholder input from clinicians on which services they most need to improve.

**Methods:**

The authors conducted a survey of primary care physicians 9–12/2021, with an emphasis on rural practices, to assess areas in which clinicians felt the greatest needs for improvement. The survey focused on primary prevention (behavior change counseling) and cancer screening, and contrasted needs for improvement for these services vs. other types of screening, and between clinicians in rural vs. non-rural practices.

**Results:**

There were 326 respondents from 4 different practice-based research networks, a wide range of practice types, 49 states and included 177 clinicians in rural settings. Respondents rated the need to improve delivery of primary prevention counseling services highest, with needs for nutrition and dietary assessment and counseling rated highest followed by physical activity and with almost no differences between rural and nonrural. Needs for improvement in cancer screenings were rated higher than non-cancer screenings, except for blood pressure screening.

**Conclusions:**

Both rural and nonrural primary care clinicians feel a need for improvement, especially with primary prevention activities. Although future research is needed to replicate these findings with different populations and other types of preventive service activities, greater priority should be given to development of practical, stakeholder informed assistance and resources for primary care to conduct primary prevention.

**Supplementary Information:**

The online version contains supplementary material available at 10.1186/s12875-022-01845-1.

## Introduction

There is a well-documented gap between research and practice in cancer prevention and control (CPC) [1], as well as most other preventive service areas [2–4]. The classic reference on this issue calculated that on average it took 17 years for 14% of the evidence to translate into practice [5]. A recent analysis indicated that there is still 15 years on average for evidence-based CPC activities to translate [6]. Of course, this is an average and for some issues and contexts such as COVID-19 or pharmaceutical interventions where there is a significant financial investment and national campaigns to accelerate adoption, this time can be significantly shorter. Primary care is expected to perform many of the activities recommended by the United States Preventive Services Task Force (USPSTF), Medicare, and professional organizations. Given competing demands and current payment priorities, often it is prevention that gets excluded from primary care [7, 8].

Clinical burden may play an even more prominent role in the implementation of newer evidence-based practices, like lung cancer screening, that lack detailed implementation roadmaps or require different documentation and processes than other risk reduction or screening practices [9]. Delivery of preventive services is especially challenging in rural settings [10] that have higher disease risks [11] fewer resources, generally fewer staff, and much less integrated care [12, 13]. Therefore, it is important to understand preventive services areas in which clinicians most need to improve and most want help, and to determine if these needs vary across different types of practices.

We conducted a clinician survey for this report as part of our NCI funded Implementation Science Center in Cancer Control (ISC3) [14]. The Colorado P50 center grant is titled Pragmatic Implementation Science Approaches to Assess and Enhance Value of Cancer Prevention and Control in Rural Primary Care. Its purpose is to develop, evaluate, implement and share pragmatic implementation science approaches reducing cost and increasing value to cancer prevention and control (CPC) in rural primary care. We focus on stakeholder engaged tailoring and adapting implementation strategies and intervention approaches to local contexts, populations, settings, and resources [15].

Knowing clinician/practice prioritized needs for improvement would inform clinician-researcher partnership development and guide efforts to offer services that are most desired by our partner primary care practices. It would also help preventive medicine researchers, program planners, and funders to understand similarities and differences in these needs across rural vs. nonrural practices as well as other clinician and practice characteristics. Some research has been conducted on physician priorities across different preventive services [16, 17] but these may not be the same as the areas in which practices most need assistance.

Thus, we sought to answer the question: are there differences among primary care clinicians in perceived need for improvement across types of preventive service activities (e.g., primary prevention behavior change counseling vs. screening); and across practice characteristics, especially rural vs. nonrural. We conducted a stakeholder informed survey of primary care clinicians to 1) identify preventive services with which clinicians would most like assistance; 2) assess relative preferences for assistance with CPC vs. other preventive service activities; and 3) investigate clinician and practice characteristics potentially related to these issues with particular focus on rural vs. nonrural differences.

## Methods

### Study setting

The survey was conducted from September to December 2021. This study was reviewed by the Colorado Multiple Institutional Review Board, approved as expedited research, and written informed consent was not required.

### Target population

A national sample of primary care clinicians was obtained from the American Academy of Family Physicians (AAFP) and several practice-based research networks (PBRNs) which are collaborating with our ISC3 Center: The National Research Network (NRN), The Virginia Ambulatory Care Outcomes Research Network (ACORN), The (Colorado) Partners Engaged in Achieving Change in Health Network (PEACHnet) and the (Colorado) High Plains Research Network (HPRN). Eligible respondents were adult primary care clinicians including doctors of medicine, doctors of osteopathic medicine, nurse practitioners and physicians’ assistants who were clinicians of record. The goal was to receive approximately 300 completed surveys to yield 80% power with a 5% Type I error rate to detect a 0.33 SD difference, or medium effect size, when comparing continuous variables between two groups of approximately equal size.

While some PBRNs were regional (limited to Colorado or Virginia), others were national (AAFP and NRN). All primary care clinicians in the entire network were invited from HPRN and PEACHnet. A random sample was obtained from the considerably larger NRN, ACORN and AAFP (rural only).

### Survey design

The survey instrument (see Appendix) covered the following conceptual areas: perceived need for improvement across various preventive services, the service with which clinicians would most like assistance, ratings of different implementation strategies (this issue is the focus of a separate paper), and physician and practice characteristics.

#### Preventive services

Prevention activities were drawn primarily from USPSTF recommendations with one exception: we added peripheral vascular disease screening because of local interest in this emerging topic. We chose services addressing 1) screening for a variety of different conditions in addition to our primary focus on cancer (hypertension, depression, alcohol use) and 2) primary prevention activities (counseling for smoking cessation, regular exercise, healthy eating). We selected 11 preventive services receiving an A or B grade from the USPSTF: as shown in Table 3 these included five cancer-related screening services; three non-cancer screening services; and four primary prevention activities (because of our interest in physical activity and nutrition, we divided the USPSTF recommendation on these activities into 2 items). Because of our ISC3 Center’s focus on cancer and stakeholder recommendations we added an item on ‘Address cancer survivorship.” Pretesting and piloting indicated that clinicians understood that they were only to rate services for ‘eligible patients,’ so we did not write out all the details concerning each recommendation (e.g., an item stated ‘high blood pressure screening’ rather than “Hypertension in Adults: Screening: adults 18 years or older without known hypertension”) [18].

#### Physician and practice characteristics

Several variables were included for descriptive purposes and as potential moderating factors. Of greatest a priori interest and a focus of many of our analyses was a classification of rural vs. nonrural clinical setting. Rural status was assessed using practice zip code reported by the respondent and the corresponding RUCA codes 4–10 [19]. Other respondent and practice characteristics included clinician specialty, gender, number of adult patients seen, years since training, size and type of practice (e.g., federally qualified health center; private practice), implementation climate [20], presence of any disease registries or prompting systems, and estimated age, insurance type, and race/ethnicity of the patient population served.

Survey pre-testing included three steps. The survey underwent internal evaluation and revision by members of the investigative team including cancer researchers (*n* = 5), local primary care clinicians (*n* = 4), and PBRN directors having large numbers of rural practices (*n* = 3). The penultimate version was pilot-tested by a group of 5 cancer researchers and practicing primary care physicians from different regions of the country. These included family doctors in Massachusetts, Missouri, Virginia and Colorado. Four out of the 5 pilot testers participated in interviews after completing the survey, and their feedback was incorporated into the final survey. Survey completion took an average of 10–12 min. Responses to feedback from pilot and pretesting included reducing the number of CPC activities, condensing and clarifying several questions, and changing the wording to reflect terms used by primary care clinicians. Feedback was then integrated and the final version was re-reviewed electronically and on hard-copy to eliminate errors and assure consistency across digital and hard-copy versions.

### Survey administration

Following Dillman’s Tailored Designed Method [21], all clinicians received an electronic cover letter endorsed by their PBRN or national organization together with the 19-item questionnaire. Those with known email addresses received an individually addressed initial survey using Qualtrics^XM^ and up to two emailed reminders. Due to rules and mandatory survey practices in different PBRNs, slightly different follow-up procedures were used after the identical initial email distribution. Those without an email address received a paper survey sent via standard mail. All email non-responders from PEACHnet and ACORN PBRNs also received a mailed survey. Each respondent was offered an incentive of $50 using RewardsLink.

Items regarding clinicians need for assistance with different preventive services were assessed using 6-point Likert-type scales. Prevention activities were displayed in random order across respondents.

### Analyses

All statistical analyses were performed using SAS software (SAS 9.4, SAS Institute, Cary, NC). Analyses were primarily descriptive and focused on means, standard deviations, and distributions for the preventive services ratings. To compare different services (repeated responses for individuals), general linear mixed effects modeling was used for continuous measures with random effect for individual respondent, adjusted for clinician characteristics that impacted results [22]. As appropriate, chi-square or ANOVA were used to evaluate potential differences associated with continuous or categorical data on physician and practice characteristics.

## Results

### Sample

We received a total of 343 surveys out of 8348 surveys sent for a response rate of 4%. We eliminated 17 ineligible respondents (6 clinicians who indicated they saw less than 1% of adult patients, 2 clinicians who practiced abroad and 9 clinicians having a degree other than M.D., D.O., P.A. or N.P.). Eligible respondents practiced in 49 different states and included 177 rural and 149 nonrural clinicians. Tables 1 and 2 summarize respondent characteristics. Most respondents were family physicians (91%) and the practices in which they worked included hospital based (39%), private (38%), federally qualified health centers (17%) and academic (13%) settings. There was a wide range of practice size (an average of 18 clinical staff, but the standard deviation exceeded the mean). An estimated 47% of respondents’ patient panels were over 50 years of age; 32% were on Medicare and 31% on Medicaid or uninsured; 66% of patients were non-Hispanic White, 15% were Latinx, 12% African American and less than 5% were Asian or American Indian/Alaskan Native; and 77% of clinicians reported having some type of registry or prompting systems for cancer prevention and control activities.

**Table 1:** Respondent Characteristics Overall

	**Overall** (*n* = 326)
Practice Location, % (n)	
Rural	54% (177)
Nonrural	46% (149)
Type of Practice, % (n)	
FQHC	16% (49)
Private Practice	38% (112)
Hospital/Health-system Owned	39% (116)
Academic	13% (40)
Other (VA, HMO)	1% (4)
Registry or Prompting System for Cancer Prevention and Control Services, % (n)	
Very Robust	31% (92)
For Some	46% (139)
No	23% (68)
Degree, % (n)	
MD	77% (251)
DO	11% (37)
NP	5% (15)
PA	4% (14)
Other (eliminated from survey)	3% (9)
Specialty, % (n)	
Family Physician	91% (293)
Internal Medicine	6% (19)
Other	4% (12)
Gender, % (n)	
Male	47% (148)
Female	53% (167)
Panel Size (patients per week), mean (SD)	73 (103)
Years from finished clinical training, years (SD)	20 (12)
Total number of clinical staff members, mean (SD)	18 (19)
Patient Age, mean % (SD)	
Percent < 18 years old	13% (11)
Percent 18–50 years old	39% (15)
Percent > 50 years old	47% (18)
Patients’ Insurance Types, mean % (SD)	
Percent Uninsured	8% (12)
Percent Medicaid	23% (18)
Percent Medicare	32% (16)
Percent Private	36% (21)
Patient’s Race and Ethnicity, mean % (SD)	
Percent White or Caucasian	66% (24)
Percent Hispanic or Latino	15% (17)
Percent Black or African American	13% (16)
Percent Asian	5% (8)
Percent Other (American Indian, Alaska Native, Native Hawaiian or Pacific Islander)	4% (9)

*FQHC* Federally Qualified Health Center, *VA* Veterans Affairs, *HMO* Health Maintenance Organization, *SD* Standard Deviation, *MD* Doctor of Medicine, *DO* Doctor of Osteopathic medicine, *NP* Nurse Practitioner, *PA* Physician’ Assistant

**Table 2:** Respondent Characteristics for Rural and Nonrural Clinicians

	**Rural** *n* = 177	**Nonrural** *n* = 149	*p*-value* (rural vs. nonrural)
Practice Location, % (n)	54%	46%	
Type of Practice ^a^, col %			
FQHC	21%	11%	**0.025**
Private Practice	31%	45%	**0.008**
Hospital/Health-system Owned	45%	32%	**0.019**
Academic	8%	20%	**0.002**
Other (VA, HMO)	4%	0%	0.179
Registry or Prompting System for Cancer Prevention and Control Services, col %			0.460
Very Robust	31%	30%	
For Some	44%	50%	
No	25%	20%	
Degree, col %			**0.008**
MD	73%	81%	
DO	10%	13%	
NP	7%	2%	
PA	5%	3%	
Other (eliminated from survey)	5%	0%	
Specialty, col %			
Family Physician	91%	91%	0.821
Internal Medicine	4%	8%	0.116
Other	5%	1%	**0.039**
Gender, col %			0.577
Male	46%	48%	
Female	54%	52%	
Panel Size (patients per week), mean (SD)	67 (45)	79 (142)	0.328
Years from finished clinical training, years (SD)	18 (12)	22 (12)	**0.005**
Total number of clinical staff members, mean (SD)	17 (19)	19 (19)	0.339
Patient Age, mean % (SD)			
Percent < 18 years old	13 (10)	14 (12)	0.780
Percent 18–50 years old	37 (16)	42 (15)	**0.018**
Percent > 50 years old	49 (19)	44 (17)	**(0.012)**
Patients’ Insurance Types, mean % (SD)			
Percent Uninsured	8 (11)	8 (13)	0.881
Percent Medicaid	25 (18)	22 (18)	0.132
Percent Medicare	36 (17)	29 (13)	**0.0002**
Percent Private	32 (19)	41 (22)	**0.0004**
Patient’s Race and Ethnicity, mean % (SD)			
Percent White or Caucasian	72 (22)	60 (24)	** < .0001**
Percent Hispanic or Latino	14 (16)	17 (19)	0.142
Percent Black or African American	8 (13)	17 (18)	** < 0.0001**
Percent Asian	4 (8)	6 (7)	**0.014**
Percent Other (American Indian, Alaska Native, Native Hawaiian or Pacific Islander)	5 (11)	3 (5)	0.084

*FQHC* Federally Qualified Health Center, *VA* Veterans Affairs, *HMO* Health Maintenance Organization, *SD* Standard Deviation, *MD* Doctor of Medicine, *DO* Doctor of Osteopathic medicine, *NP* Nurse Practitioner, *PA* Physician’ Assistant

^*^Chi-Square for comparison between rural and nonrural (*p* < 0.05 in bold)

^a^Type of Practice: percentages within rural and nonrural exceed 100% because respondents could select all that applied

### Needs for improvement

Table 3 summarizes the level of reported need for improvement for the preventive services included. Because of our focus on CPC and rural primary care, we present these ratings by rural and nonrural clinicians and have categorized the 13 preventive service activities into primary prevention, cancer screening, non-cancer screening, and cancer survivorship.

**Table 3:** Means and Standard Deviations on Ratings of Need for Improvement by CPC Activity and Rural/Nonrural Clinicians

	**Overall** ***n* = 312**		**Rural** ***n* = 165**		**Non-rural** ***n* = 147**	
	**Mean**	**SD**	**Mean**	**SD**	**Mean**	**SD**
**Primary Prevention Activities Composite**	4.33	1.02	4.31	0.96	4.35	1.09
Physical activity assessment and counseling	4.48	1.21	4.43	1.20	4.53	1.22
Alcohol use assessment and counseling	4.28	1.23	4.23	1.17	4.33	1.29
HPV discussion and vaccine	3.88	1.45	3.87	1.44	3.88	1.46
Nutritional/dietary assessment and counseling	4.63	1.22	4.59	1.23	4.66	1.21
Tobacco use assessment and cessation counseling	4.38	1.44	4.40	1.37	4.35	1.52
**Cancer Screening Composite**	4.05	1.17	4.01	1.14	4.10	1.20
Lung cancer screening	4.09	1.26	4.04	1.26	4.16	1.26
Colorectal cancer screening	4.27	1.32	4.23	1.32	4.32	1.31
Cervical cancer screening	3.79	1.42	3.73	1.44	3.86	1.39
Breast cancer screening	4.05	1.38	4.04	1.33	4.05	1.45
**Non-cancer Screening Composite**	3.68	1.21	3.66	1.17	3.70	1.26
Screening for lipid disorders	3.66	1.48	3.64	1.47	3.68	1.49
High blood pressure screening	4.00	1.61	3.92	1.57	4.08	1.67
Screening for peripheral artery disease with ankle brachial index	3.37	1.31	3.41	1.31	3.33	1.32
**Other**						
Address cancer survivorship (e.g., survivorship care plans)	3.72	1.35	3.74	1.39	3.70	1.31

*CPC* Cancer Prevention and Control, *SD* Standard Deviation, *HPV* Human Papillomavirus

Clinicians felt the greatest need for improvement with primary prevention behavior change counseling activities; in fact, the three individual activities rated as having the greatest need were assessment and counseling for: nutrition and dietary behaviors (mean of 4.63 of 6 possible); physical activity (M = 4.48); and tobacco use (M = 4.38). Ratings of need for improvement for the composite of the five primary prevention activities was significantly higher than either cancer or non-cancer screening composites (*p* < 0.0001; Table 3). Although the differences were not quite as strong, the rated need for improvement with cancer screening activities was generally higher than that for non-cancer screening (M = 4.05 vs. 3.68; *p* < 0.0001). Among the screening activities, need for improvement was highest among colorectal cancer (M = 4.27) followed by breast cancer screening (M = 4.05) and lowest for PAD (M = 3.37) and lipid screening (M = 3.66). Cancer survivorship was among the areas rated as having the least need for improvement (M = 3.72).

As detailed in Table 3, there were few differences between ratings from rural and nonrural clinicians and composite ratings were very similar for these groups. In general, need for improvement was rated slightly and nonsignificantly lower in rural than nonrural settings. To further assess potential impacts of rurality, we conducted sub analyses between physicians in isolated and small rural settings (RUCA codes 10.0, 10.2, 10.3, 10.4, 10.5, 10.6 and 7.0, 7.2, 7.3, 7.4, 8.0, 8.2, 8.3, 8.4, 9.0, 9.1, 9.2; *n* = 69) and those in larger rural settings (RUCA codes 4.0, 4.2, 5.0, 5.2, 6.0, 6.1; *n* = 88). These analyses did not reveal any significant differences between these groups on any of the 13 prevention activities; and the same two prevention activities- diet/nutrition and physical activity were rated as the areas most in need of assistance for both groups.

**Fig. 1:**
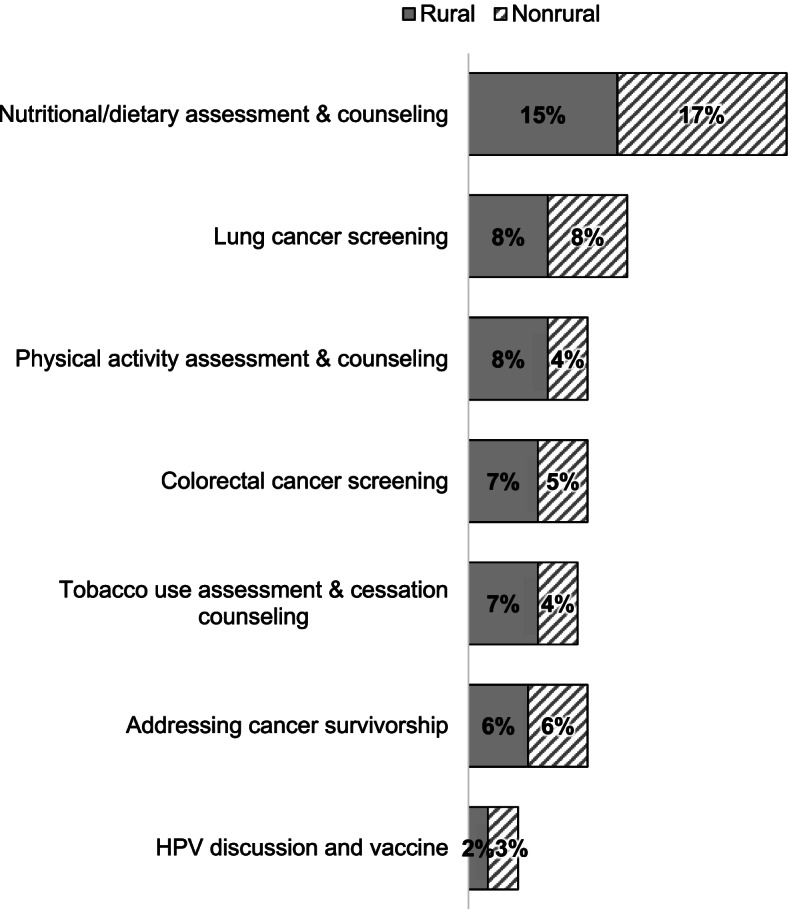
Percent of time clinicians chose each of 7 CPC activities as the area they would most like help Implementing in their practice (*n* = 326). CPC = Cancer Prevention and Control; HPV = Human Papillomavirus

Because of our interest in CPC, the survey asked respondents to select one item among the seven primary prevention and cancer screening activities they would most like help implementing (see Appendix 1). Once again nutrition/dietary counseling was selected the most frequently by both rural and nonrural respondents, followed by lung cancer screening and physical activity assessment and counseling (Fig. 1: 32% vs. 16% and 12% respectively).

#### Subgroup and moderator analyses

Using the summary categories of primary prevention, cancer screening and non-cancer screening, we evaluated relationships between these composite ratings and various clinician/practice characteristics. With two exceptions there were no significant differences on ratings associated with clinician or practice characteristics. Female respondents reported a higher need for assistance with primary prevention than did male clinicians. Physician assistants reported a higher need for assistance with noncancer screenings than did MDs, DOs or NPs. Inclusion of these variables as moderators in analyses did not alter results regarding rural-nonrural differences.

## Discussion

This survey of primary care clinicians elicited respondent perceptions about a range of primary prevention, cancer screening, and non-cancer related screening activities. Although not a representative survey, respondents came from four PBRNs, 49 states and a wide range of practice types and sizes in both rural and nonrural settings. Overall, there was a higher rated need for improvement of delivery of primary prevention activities than various types of screening. Among screening services, respondents reported a greater need for assistance with cancer screenings than non-cancer related screenings.

We were surprised that there was such high perceived need for improvement with primary prevention counseling, especially given that most of these activities are not reimbursed, and often not part of clinical ‘dashboards or quality metrics [23] apart from tobacco cessation counseling. It may also be that the importance of primary prevention is recognized among physicians, but since there is little or no reimbursement, they see greater need for assistance if they are going to be able to address these issues than for care issues for which they are held accountable and receive reimbursement. It should be emphasized that these ratings were not of the relative priority [17] or ranked importance among this list of preventive services [24], but rather of the *need for improvement*. It might be that some screening services are felt to be of highest priority, but that clinician’s resources and confidence in delivering these services are also high, and thus these activities were not rated highly on need for improvement.

While there are considerable data on the relative benefit of and physician ratings of different preventive services [24–26] and some research on the delivery of different types of primary and secondary prevention activities [27–29], we are not aware of research on clinician perception of need for improvement of different services. Given the importance of primary prevention in terms of population health [30], the challenges of delivering quality primary prevention in most primary care settings [8] and our data on rated need for improvement, greater priority should be given to development of practical, stakeholder informed, broadly applicable assistance and resources for primary prevention.

There was a moderately high level of rated need for improvement across preventive service activities, but to our surprise, almost no differences between clinicians in rural and nonrural settings. Given the documented higher levels of risk, poorer health, greater social determinants of health challenges and generally fewer resources in rural settings [10–13], we expected there to be greater needs for improvement among rural clinicians. This lack of differences was not explained by any of several potential moderating variables including patient populations, staffing patterns, size or types of practices. There may be other factors that moderated our results that were not assessed, or it may be that rural practitioners are used to ‘doing it on their own’ and despite having generally fewer resources, do not perceive any greater need for assistance.

Two preventive services deserve additional comment. Despite its relatively recent addition to USPSTF recommended practices and very low uptake nationally, respondents did not rate lung cancer screening as a priority area of need. Given the disproportionate lung cancer burden experienced in rural areas, the lack of rural-nonrural differences was somewhat unexpected. This could be due multiple factors, including low awareness of lung cancer screening data and policy among primary care clinicians [31], concerns about the merits of lung cancer screening [32, 33], or the complexity of the lung cancer screening process [9]. Second, screening for PAD was rated as having the lowest need for improvement, serving as a type of validity check as it was the only non USPSTF recommended activity.

This report has several strengths and some moderate limitations. Its strengths include the moderately large sample size and especially the good-sized sample of rural primary care clinicians; a wide variety of practice types, clinician and patient panel characteristics; the random order of item presentation across respondents to control for potential order effects; inclusion of a wide range of USPSTF preventive services; and comparison of cancer to non-cancer screening activities as well as of primary prevention to screening activities. We were also able to conduct some subgroup comparisons, especially those relevant to rural-nonrural differences for which there are often insufficient sample sizes to conduct such analyses.

Key limitations include the relatively low survey return rate despite following many best survey research practices recommended by Dillman and others [21, 34], inclusion of signed, strong letters of support from PBRN leaders and a $50 stipend. This return rate was likely due at least in part to the challenges of coping with COVID-19 and in some cases even concerns about the continued existence of the practices of the clinicians surveyed. Consequently, this is not a representative national sample; is composed of primarily family physicians; and does not represent the perspectives of internal medicine physicians or other practitioners (e.g., PAs and NPs who deliver many services in rural and low resource practices).

Although we have speculations concerning why the observed pattern of results was obtained, without supporting qualitative data or experimental tests of such interpretations we cannot be confident in these explanations. For example, it would be of interest to know how many of the physicians in this study have regular access to a dietician or behavioral health clinician. There was also considerable variability across practices, suggesting that there may be other unmeasured factors that influenced our results. Finally, although we measured and evaluated factors such as type and size of practice, implementation climate, and race/ethnicity, our survey did not directly address detailed or structural health equity issues that may have influenced results.

Our future work with our primary care practice partners as part of our COISC3 center will be informed by the observed preferences and areas of need identified by these stakeholders – rather than our developing programs and approaching practices about topics for which clinicians do not perceive a need. We are also providing feedback to our partner PBRN organizations to help them with their planning and educational activities. A final implication is that work should be conducted to provide resources, training and ongoing support for primary care offices to address primary prevention issues. An initial step would be to identify the specific content, modalities, format, and other features of such resources that will allow primary care practices to integrate them into their workflow.

## Conclusions

This study identified a higher perceived need for improvement with primary prevention counseling activities among primary care clinicians, especially for assessment and counseling for dietary behaviors and physical activity, than for a variety of other recommended USPSTF screening activities. Our study identified a need for outside assistance to address the highest rated prevention activities for improvement by primary care physicians. Despite numerous differences on practice characteristics between rural and nonrural practices (Table 2) there were almost no differences on rated needs between clinicians in rural and nonrural settings. Future research is needed to replicate these findings with different settings and populations, including other types of preventive service activities.

## Supplementary Information

**Additional file 1: Appendix 1.** Survey on Primary Care Priorities for Prevention Activities.

## Data Availability

The datasets during and/or analyzed during the current study available from the corresponding author on reasonable request.

## Abbreviations

CPC: Cancer prevention and control
ISC3: Implementation Science Center in Cancer Control
COISC3: Colorado P50 center grant, Pragmatic Implementation Science Approaches to Assess and Enhance Value of Cancer Prevention and Control in Rural Primary Care
AAFP: American Academy of Family Physicians
PBRNs: Practice-based research networks
ERIC: Expert Recommendations for Implementing Change
M: Mean
HPV: Human papillomavirus
USPSTF: United States Preventive Services Task Force
MD: Doctor of Medicine
DO: Doctor of Osteopathic medicine
PA: Physician’s Assistant
NP: Nurse Practitioner
