# Supplementary material for: Priorities for improvement across cancer and non-cancer related preventive services among rural and non-rural clinicians
Source: BMC Prim Care. 2022 Sep 9;23:231. doi: 10.1186/s12875-022-01845-1 (PMC9462636; doi:10.1186/s12875-022-01845-1)
Supplement: Supplementary file 1 — Additional file 1: Appendix 1. Survey on Primary Care Priorities for Prevention Activities. [file 12875_2022_1845_MOESM1_ESM.docx]

**Appendix 1: Survey on Primary Care Priorities for Prevention Activities**

**We would like to understand how you think about and prioritize prevention and screening for different chronic illnesses among your adult patients. This survey should take approximately 10 minutes. You will be compensated with a $50 gift card, or you may elect to donate the $50 to one of several charities. To be eligible for the incentive, you need to be one of the following: Doctor of Medicine (MD), Doctor of Osteopathic Medicine (DO), Nurse Practitioner (NP), or a Physician's Assistant (PA).**

**Your responses will be confidential. Results will be reported only in summary fashion. You, and if relevant, your practice-based research network will receive summary results of the survey.**

*This survey is being conducted as part of the Pragmatic Implementation Science Approaches to Assess and Enhance Value of Cancer Prevention and Control in Rural Primary Care Project funded by The National Cancer Institute. The research study is based at the University of Colorado | Anschutz Medical Campus. Participation in this survey is completely voluntary. By answering the questions and submitting the questionnaire you are providing consent and permission to use the information shared. If you have questions, you can contact Bryan Ford at bryan.ford@cuanschutz.edu or 303.724.3422. If you have questions about your rights as a participant, you can call the COMIRB (the responsible Institutional Review Board) at 303-724-1055. We believe this research study presents no risk to all participants; if you do not want to respond to any question, simply skip it. There may be risks the researchers have not thought of. This study is not designed to benefit you directly. The data we collect will be used for this study but may also be important for future research. Your data may be used for future research or distributed to other researchers for future study without additional consent if information that identifies you is removed from the data.*

1. **What is your degree?**

M.D. D.O. N.P. P.A. Other

Please stop here! Unfortunately, you are not eligible to fill out the rest of the survey due to our specific criteria.

1. **What is your practice specialty?** *(Please check ALL that apply)*

Family Medicine

Internal Medicine

Other *(please specify)*: ________________________

1. **Please type the year in which you finished your clinical training:** ____________
2. **Please select the gender with which you most identify:**

Man Woman Non-binary

1. **Please provide the zip code where your practice is located: ________________**
2. **How important is it for your practice to IMPROVE in each of the following areas?**

| **Activity** | **Not at all important** | **Low importance** | **Somewhat important** | **Moderately important** | **Very important** | **Extremely important** |
| --- | --- | --- | --- | --- | --- | --- |
| 1. Address cancer survivorship (e.g., survivorship care plans) |  |  |  |  |  |  |
| 1. Lung cancer screening |  |  |  |  |  |  |
| 1. Screening for lipid disorders |  |  |  |  |  |  |
| 1. Physical activity assessment and counseling |  |  |  |  |  |  |
| 1. Colorectal cancer screening |  |  |  |  |  |  |
| 1. High blood pressure screening |  |  |  |  |  |  |
| 1. Alcohol use assessment and counseling |  |  |  |  |  |  |
| 1. Cervical cancer screening |  |  |  |  |  |  |
| 1. HPV discussion and vaccine |  |  |  |  |  |  |
| 1. Nutritional/dietary assessment and counseling |  |  |  |  |  |  |
| 1. Breast cancer screening |  |  |  |  |  |  |
| 1. Screening for peripheral artery disease with ankle brachial index |  |  |  |  |  |  |
| 1. Tobacco use assessment and cessation counseling |  |  |  |  |  |  |

1. **From the list above, please type in your top 3 priorities. (These do not have to be in ranked order.)**

**___________________ ___________________ ___________________**

**7.B. With regard to ________, do you want any training, tools or other support to facilitate this activity?**

No, not interested

No, but possibly in the future

Yes, possibly within the next six months

Yes, within the next 30 days

- Yes, we are currently working on this and would consider additional resources

**7.C. With regard to ________, do you want any training, tools or other support to facilitate this activity?**

No, not interested

No, but possibly in the future

Yes, possibly within the next six months

Yes, within the next 30 days

- Yes, we are currently working on this and would consider additional resources

**7.A. With regard to ________, do you want any training, tools or other support to facilitate this activity?**

No, not interested

No, but possibly in the future

Yes, possibly within the next six months

Yes, within the next 30 days

- Yes, we are currently working on this and would consider additional resources

1. **Which of the following 7 cancer prevention activities would you most like help with implementing consistently in your practice?** *(Please select 1 activity and type it in)*

- For each strategy, please rate both its **FEASIBILITY** and its **IMPACT**
  - By **FEASIBLE**, we mean how easy it would be to conduct this strategy in your practice.
  - By **IMPACT**, we mean the effect this strategy would have on facilitating consistent delivery in your practice.

1. Colorectal Cancer screening
2. Nutritional/dietary assessment and counseling
3. Tobacco use assessment and cessation treatment or referral
4. Lung Cancer Screening
5. Physical activity assessment and counseling
6. HPV Vaccination
7. Address cancer survivorship (e.g. survivorship care plan)

**Activity: ______________________ ___________** *(please type in from a-g above)*

**8.A.**  **For the activity you selected, please rate each of the following strategies on their (1) FEASIBILITY and (2) IMPACT for helping your practice team conduct this activity consistently.**

| **Activity:** ____________________________ | **FEASIBILITY**  (How easily could this be conducted in your setting?) | | | | | |
| --- | --- | --- | --- | --- | --- | --- |
| **Strategy for Assistance** | **Not Feasible** | **A Little Feasible** | **Somewhat Feasible** | **Moderately Feasible** | **Very Feasible** | **Extremely Feasible** |
| 1. Have a practice facilitator or coach |  |  |  |  |  |  |
| 1. Build a health information technology tool (e.g., EHR reminder or decision aid) |  |  |  |  |  |  |
| 1. Assess and redesign clinic workflow |  |  |  |  |  |  |
| 1. Use audit and feedback or some type of periodic data reporting |  |  |  |  |  |  |
| 1. Adapt evidence-based intervention (or guideline) to our practice |  |  |  |  |  |  |
| 1. Use a quality improvement approach |  |  |  |  |  |  |
| 1. Refer patients to community resources (e.g., WIC, YMCA, Quitline) |  |  |  |  |  |  |
| 1. Training and education for practice staff |  |  |  |  |  |  |
| 1. Engage patients to help create an individually tailored action plan |  |  |  |  |  |  |

| **Activity:** ____________________________ | **IMPACT**  (How effective would this strategy be IF it is implemented in your practice?) | | | | | |
| --- | --- | --- | --- | --- | --- | --- |
| **Strategy for Assistance** | **No impact** | **Low Impact** | **Some Impact** | **Moderate Impact** | **High Impact** | **Very high Impact** |
| 1. Have a practice facilitator or coach |  |  |  |  |  |  |
| 1. Build a health information technology tool (e.g., EHR reminder or decision aid) |  |  |  |  |  |  |
| 1. Assess and redesign clinic workflow |  |  |  |  |  |  |
| 1. Use audit and feedback or some type of periodic data reporting |  |  |  |  |  |  |
| 1. Adapt evidence-based intervention (or guideline) to our practice |  |  |  |  |  |  |
| 1. Use a quality improvement approach |  |  |  |  |  |  |
| 1. Refer patients to community resources (e.g., WIC, YMCA, Quitline) |  |  |  |  |  |  |
| 1. Training and education for practice staff |  |  |  |  |  |  |
| 1. Engage patients to help create an individually tailored action plan |  |  |  |  |  |  |

**Practice Characteristics**

1. **Please indicate your level of agreement with each of the following statements regarding clinical staff at your practice.** *(Please check one box for each row)*

| ***“Clinical staff at my practice …”*** | **Strongly Disagree** | **Disagree** | **Agree** | **Strongly Agree** |
| --- | --- | --- | --- | --- |
| 1. … are expected to discuss evidence-based cancer prevention and screening with a certain number of patients. |  |  |  |  |
| 1. … are expected to help our practice meet its goals for implementing evidence-based cancer prevention and screening. |  |  |  |  |
| 1. … get the support they need to identify potentially eligible patients for evidence-based cancer prevention and screening. |  |  |  |  |
| 1. … get the support they need to discuss evidence-based cancer prevention and screening with eligible patients. |  |  |  |  |
| 1. … receive recognition for discussing evidence-based cancer prevention and screening with eligible patients. |  |  |  |  |
| 1. … receive appreciation for discussing evidence-based cancer prevention and screening with eligible patients. |  |  |  |  |

**If you work in more than 1 practice or clinic location, please respond for the practice where you spend the majority of your work time.**

1. **Please estimate the total number of clinical staff members in your practice.**  ___________
2. **How would you describe your practice?** *(Please check ALL that apply)*

Federally Qualified Health Center

Private Practice

Health Maintenance Organization

Hospital or Health System Owned

VA

Academic

1. **Do you have any registries or prompting systems regarding cancer prevention and control activities?**

Yes, very robust Yes, for some No

1. **Do you have reliable internet access with sufficient bandwidth/speed at your practice?**

Yes No

1. **Going forward, what percentage of your patient visits do you anticipate having virtual visits (video or telephone)? Please ESTIMATE:** ________________

______________

1. **On average, how many adult patients do you see per week?** ________________
2. **What is the approximate percentage of patients in your practice in each of the following categories? Please give your best ESTIMATE:**

| **Age** | **% of patients** |
| --- | --- |
| 1. < 18 years old |  |
| 1. 18-50 years old |  |
| 1. > 50 years old |  |

| **Type of Insurance** | **% of patients** |
| --- | --- |
| 1. Uninsured |  |
| 1. Medicaid |  |
| 1. Medicare |  |
| 1. Private insurance |  |

| **Race and Ethnicity (may not add to 100%)** | **% of patients** |
| --- | --- |
| 1. Hispanic or Latino |  |
| 1. Black or African American |  |
| 1. Asian |  |
| 1. American Indian or Alaska Native |  |
| 1. Native Hawaiian or Pacific Islander |  |
| 1. White or Caucasian |  |

1. **Please provide any other comments or feedback about factors related to issues of cancer prevention and control in your practice:**
2. **If selected, would you be willing to participate in a brief paid interview as a follow-up to this survey?**

Yes

**What is your preferred mode of contact?**

Email (please provide your email address): ____________________________________

Phone (please provide your phone number): **___________________________________**

No

1. **We appreciate your time in taking this survey. You will receive an email from noreply@tangocard.com with a $50 RewardLink that you can redeem to a retailer or donate to a charity. If you would like to decline payment for this survey, you can opt out below.**

I would like to receive the $50

**Please write your preferred email address to receive the $50 RewardLink:**

**__________________________________________________**

I would like to decline the $50

**Thank you for participating**.

Please address any questions to and return the survey in the enclosed stamped envelope addressed to:

Elise Robertson, MA / National Research Network

11400 Tomahawk Creek Pkwy, Leawood, KS 66211
